# Supplementary material for: Patterns of Intron Gain and Loss in Fungi
Source: PLoS Biol. 2004 Nov 30;2(12):e422. doi: 10.1371/journal.pbio.0020422 (PMC532390; doi:10.1371/journal.pbio.0020422)
Supplement: Table S1 — Also available at http://genes.mit.edu/NielsenEtAl/. (4.3 MB ZIP). [file pbio.0020422.st001.zip › NielsenEtAl/html/1227.html]

AN1921.1.NCU04802.1.MG08896.1.FG09281.1


```
 CLUSTAL W (1.82) Multiple Sequence Alignments - Introns Inserted


Sequence 1: AN1921.1	235 aa
Sequence 2: NCU04802.1	234 aa
Sequence 3: MG08896.1	234 aa
Sequence 4: FG09281.1	235 aa
Alignment Length: 236 aa
Number Identitical Residues: 107 aa
Alignment Score (without introns) 5754


MG08896.1 	MVADALVYHPTVSHYLKFVATTV~GRDKVLRVIQYFARFYAWYLFRTNGTPGEIAPWAAI
NCU04802.1	MVADALVYHPAVSHYLKFVATTV~GRDKLLRLLQYFARFYAWYLLRTNKPASAIQPWETM
FG09281.1 	MVADAVIYHPTVAHYLRYMATTL~GRDKLMRVFQYFARFYAWYLLRGNATADKVAPWNAL
AN1921.1  	MVADALVYHPALAHYLRFVATTV1GRDKLLRTLQYFSRFYAWYLYRTNQPQTAIAPYNAV
          	*****::***:::***:::***: ****::* :***:******* * * .   : *: ::

MG08896.1 	KKQFGLVRKFMRVGKNVEHLRAAAVAADAKT-ADPVVRYTTVGRQLGYAGYLTFDAATVL
NCU04802.1	KKQFGLVRKVMRAGKNVEHIKAAAVAADSKN-GDPILRYLAVGRQLGYAGYLTFDLGTLL
FG09281.1 	KKQFGLFRKVFRAGKFVEHLKAAATASDSKS-MDPVLKYTQVGRQLGYAGYLACDSLTIP
AN1921.1  	KKQFGTTRKILRIGKFIEHLKAAALAADNKGPIDPVLRYLAIGRQLGYAGYLSLDTVTVV
          	*****  **.:* ** :**::*** *:* * . **:::*  :**********: *  *: 

MG08896.1 	DAAGIRPSKNAARLGREAFRFWAFGLTFSIVGQLYALWRLKEREARVDRKEGEGVVEGKR
NCU04802.1	DATGIRKTESAKRLARESQRFWVMGLSCSVIAQAYTLWRLRQREARVDRKEGEGVVEAKR
FG09281.1 	HAAGIKTWKHATRMQHEAYRSWAVGIAFSILGQLYTLRQLSARASKVDLKEGEGVVESKT
AN1921.1  	DVIGFRKLASVKRLQDSAYRAWFSGLACSVLAGVYTLWRLSQKQKTLDLKEGEGVVEAKK
          	.. *::    . *:  .: * *  *:: *::.  *:* :*  :   :* ********.* 

MG08896.1 	IAI2ERAAARTQLISDLCDLTIPTSALGWIP-FDDGIVGIAGTVSSLIGVMAQWKKTA
NCU04802.1	IEV2ERAASRIQLTSDLCDLTVPLSALAWVN-FDDGIVGLAGTLSSLLGIYTQWKKTA
FG09281.1 	ISI2ERAAAKKQLLCDVCDILSFIGGLGYNKYLDDGVVGLTGTLSSIIGVYTQWKKTA
AN1921.1  	LEK2ERSAARIQLISDLCDLTVPVSAIGLAN-LDDGLVGIAGTISSLIGVWSQWRKTA
          	:   **:*:: ** .*:**:    ..:.    :***:**::**:**::*: :**:***
```
